# Supplementary material for: Empirical methods for controlling false positives and estimating confidence in ChIP-Seq peaks
Source: BMC Bioinformatics. 2008 Dec 5;9:523. doi: 10.1186/1471-2105-9-523 (PMC2628906; doi:10.1186/1471-2105-9-523)
Supplement: Additional File 1 — A variety of html documents from the USeq web site detailing the available applications, their best usage, output file type descriptions, command line menus, etc. [file 1471-2105-9-523-S1.zip › USeqUserGuides/JavaDoc.zip!/JavaDoc/trans/cel/package-tree.html]

trans.cel Class Hierarchy


|  |  |  |
| --- | --- | --- |
| **Overview** \| **Package** \| Class \| Use \| **Tree** \| **Deprecated** \| **Index** \| **Help** | |  |
| **PREV**   **NEXT** | **FRAMES**    **NO FRAMES**     **All Classes** |  |


---


## Hierarchy For Package trans.cel

**Package Hierarchies:**: All Packages

---

## Class Hierarchy

- java.lang.Object
  - trans.cel.**AverageACels**- trans.cel.**AveragePCels**- trans.cel.**CelFileConverter**- trans.cel.**CelLine** (implements java.lang.Comparable<T>)- trans.cel.**CelMapper**- trans.cel.**CelPHistogram**- trans.cel.**CelProcessor**- trans.cel.**ChromosomeIndex**- trans.cel.**ComparatorMultiSetQuantile** (implements java.util.Comparator<T>)- trans.cel.**ComparatorQuantile** (implements java.util.Comparator<T>)- trans.cel.**ControlStats** (implements java.io.Serializable)- trans.cel.**ConvertTVSToCelps**- trans.cel.**MakeChromosomeSets**- trans.cel.**MultiSetQuantile** (implements java.io.Serializable)- trans.cel.**Quantile**- trans.cel.**QuantileNormalization**- trans.cel.**RotateCelFile**- trans.cel.**SplineScalar**- trans.cel.**StatIntensityFeatureArray**

---


|  |  |  |
| --- | --- | --- |
| **Overview** \| **Package** \| Class \| Use \| **Tree** \| **Deprecated** \| **Index** \| **Help** | |  |
| **PREV**   **NEXT** | **FRAMES**    **NO FRAMES**     **All Classes** |  |


---
